# Distinct Lineages of Feline Parvovirus Associated with Epizootic Outbreaks in Australia, New Zealand and the United Arab Emirates

**DOI:** 10.3390/v11121155

**Published:** 2019-12-13

**Authors:** Kate Van Brussel, Maura Carrai, Carrie Lin, Mark Kelman, Laura Setyo, Danielle Aberdein, Juliana Brailey, Michelle Lawler, Simone Maher, Ildiko Plaganyi, Emily Lewis, Adele Hawkswell, Andrew B. Allison, Joanne Meers, Vito Martella, Julia A. Beatty, Edward C. Holmes, Nicola Decaro, Vanessa R. Barrs

**Affiliations:** 1 Sydney School of Veterinary Science, Faculty of Science, University of Sydney, Camperdown, NSW 2050, Australia; kate.vanbrussel@sydney.edu.au (K.V.B.); maura.carrai@sydney.edu.au (M.C.); clin5923@uni.sydney.edu.au (C.L.); kelmanscientific@gmail.com (M.K.); laura.setyo@sydney.edu.au (L.S.); jbra0176@uni.sydney.edu.au (J.B.); simone.maher@sydney.edu.au (S.M.); julia.beatty@sydney.edu.au (J.A.B.); 2 School of Veterinary Science, Massey University, Palmerston North 4410, New Zealand; D.aberdein@massey.ac.nz; 3 RSPCA NSW, Yagoona, NSW 2199, Australia; 4 Lort Smith Animal Hospital, North Melbourne, Victoria 3051, Australia; iplaganyi@lortsmith.com; 5 SPCA Wellington, Wellington 6021, New Zealand; ylimesiwel@gmail.com (E.L.); adele.hawkswell@spca.nz (A.H.); 6 Department of Comparative, Diagnostic, and Population Medicine, College of Veterinary Medicine, University of Florida, Gainesville, FL 32610, USA; aallison1@ufl.edu; 7 School of Veterinary Science, The University of Queensland, Gatton, QLD 4343, Australia; j.meers@uq.edu.au; 8 Department of Veterinary Medicine, University of Bari, Valenzano, 70121 Bari, Italy; vito.martella@uniba.it (V.M.); nicola.decaro@uniba.it (N.D.); 9 Marie Bashir Institute for Infectious Diseases and Biosecurity, Charles Perkins Centre, School of Life & Environmental Sciences and Sydney Medical School, The University of Sydney, Sydney, NSW 2006, Australia

**Keywords:** carnivore protoparvovirus, feline panleukopenia, canine parvovirus, outbreaks, infectious enteritis

## Abstract

Feline panleukopenia (FPL), a frequently fatal disease of cats, is caused by feline parvovirus (FPV) or canine parvovirus (CPV). We investigated simultaneous outbreaks of FPL between 2014 and 2018 in Australia, New Zealand and the United Arab Emirates (UAE) where FPL outbreaks had not been reported for several decades. Case data from 989 cats and clinical samples from additional 113 cats were obtained to determine the cause of the outbreaks and epidemiological factors involved. Most cats with FPL were shelter-housed, 9 to 10 weeks old at diagnosis, unvaccinated, had not completed a primary vaccination series or had received vaccinations noncompliant with current guidelines. Analysis of parvoviral VP2 sequence data confirmed that all FPL cases were caused by FPV and not CPV. Phylogenetic analysis revealed that each of these outbreaks was caused by a distinct FPV, with two virus lineages present in eastern Australia and virus movement between different geographical locations. Viruses from the UAE outbreak formed a lineage of unknown origin. FPV vaccine virus was detected in the New Zealand cases, highlighting the difficulty of distinguishing the co-incidental shedding of vaccine virus in vaccinated cats. Inadequate vaccination coverage in shelter-housed cats was a common factor in all outbreaks, likely precipitating the multiple re-emergence of infection events.

## 1. Introduction

Feline panleukopenia (FPL) is a highly contagious and often fatal disease characterised by acute severe enteritis, severe dehydration and sepsis due to lymphoid depletion and pancytopenia [1]. FPL is usually associated with infection by feline parvovirus (FPV), a member of the genus *Protoparvovirus* (formerly *Feline panleukopenia virus*). *Protoparovirus* is one of eight genera of vertebrate viruses within the subfamily *Parvovirinae* of the family *Parvoviridae.* Collectively, FPV and canine parvovirus (CPV), along with associated variants found in various carnivore species such as mink and raccoons, constitute the species *Carnivore protoparvovirus 1* [2].

Until the 1980s, FPV was the only reported viral cause of FPL in cats. FPV is able to infect cats by first binding to the feline transferrin receptor (fTfR) expressed on the surface of cells, followed by clathrin-mediated endocytosis to initiate infection [3]. Canine parvovirus CPV-2 emerged in the late 1970s and was initially unable to infect cats, as it could not bind to the fTfR [3]. However, infectivity for feline cells was acquired soon after by the genetic variant CPV-2a, which emerged in 1979 and replaced CPV-2 [4]. The ability to infect cats has also been retained by subsequent antigenic variants of CPV-2a, termed CPV-2b and CPV-2c, which only differ from CPV-2a at a single amino acid position (VP2 426). These and other antigenic variants of CPV can cause FPL in both naturally acquired and experimental infections of cats [5,6,7,8].

In contrast to parvoviral enteritis in dogs, estimated to cause 20,000 cases per year in Australia, clinical cases of FPL have rarely been diagnosed in Australia since the mid-1970s, and there have been no reports of FPL outbreaks for over 40 years [9]. In 2014, FPL re-emerged in eastern Australia, and subsequent outbreaks occurred between 2015 and 2018 in several locations in this region. Similarly, outbreaks of FPL occurred in New Zealand (NZ) between 2016 and 2018, as well as in the United Arab Emirates (UAE) in 2017, with no outbreaks of FPL reported in either country in recent decades, likely due to the widespread use of FPL vaccines.

The contemporaneous re-emergence of FPL in geographically distinct settings long considered FPL-free has raised questions as to whether virus-related or other unknown risk factors played a role in the observed FPL outbreaks. Herein, case data and clinical samples from 989 and 113 cats, respectively, were analysed to identify the lineages of *Carnivore protoparvovirus 1* responsible for the outbreaks of FPL in Australia (2014 to 2018), the UAE (2017) and NZ (2017–2018) and evaluate epidemiological factors associated with these outbreaks, including vaccination status.

## 2. Materials and Methods

### 2.1. Retrospective Case Data Analysis

Inclusion criteria for cases of FPL were (i) clinical signs typical of FPL (lethargy, fever, hypothermia, anorexia, vomiting, diarrhoea and/or sudden death) and a positive confirmatory test (faecal CPV antigen test or PCR) or (ii) clinical signs typical of FPL in a cat from a shelter with a confirmed contemporaneous outbreak of FPL.

Australian case data were extracted from a national online companion animal disease surveillance-reporting database launched in January 2010 [10] and from the medical records of animal shelters and/or veterinary hospitals in outbreak regions for the period 1 January 2014 to 31 August 2018. Data recorded included case occurrence date, shelter location, shelter post code, age at diagnosis, sex, post code of owner residence or where found as a stray before entry into shelter, clinical signs at presentation, date of last vaccination, vaccination type (inactivated or modified live virus (MLV) vaccine), time interval between last vaccination and onset of clinical signs (days), method of diagnosis and outcome. Data obtained were searched to identify cases for which serial monitoring of faecal shedding of FPV or CPV using qPCR testing had been performed. Information about animal movements, biosecurity and vaccination protocols was obtained from shelter veterinarians and/or managers.

New Zealand case data were obtained from one shelter, comprising summary data of case diagnoses for two FPL outbreaks occurring between 2016 and 2018 as well as individual data for 9 cases in which clinical samples were available for PCR and sequencing. Individual case data for UAE cases were available during the period of the outbreak in 2017.

Data were analysed using Microsoft Excel^®^ for Mac Version 16.16.15 and Statistix^®^ version 10.0 (Analytical Software, Tallahassee, FL, USA). Descriptive statistics were generated for FPL case numbers, age at disease diagnosis, interval between vaccination and disease and interval between admission and disease. Frequency distributions were created for age at disease diagnosis. Mapping and geospatial analysis was performed with ArcGIS^®^ version 10.2 (Ersi, Redlands, CA, USA).

### 2.2. Prospective Sample Collection

Residual diagnostic faecal samples or tissue (intestine or mesenteric lymph node) obtained post-mortem were collected prospectively in Australia from 7 April 2015 until 30 August 2018 and from the UAE and New Zealand during suspected outbreaks of FPL in 2017. In addition, a stored faecal sample from a cat diagnosed with FPL in the first outbreak in Australia in 2014 was obtained for study.

### 2.3. DNA Extraction and PCR

DNA was extracted for sequencing of the *Carnivore protoparvovirus* 1 VP2 gene from tissue using the Qiagen DNeasy Blood and Tissue Kit (Qiagen, Hilden, Germany) or from faecal samples using the QIAamp Fast DNA Stool Mini Kit (Qiagen, Germany) or by homogenisation, boiling and centrifugation, as previously described [11]. The extracted DNA was amplified by conventional PCR using 5 u/µL of My Taq HS Red DNA polymerase (Bioline, USA), 1–90 ng of DNA, 1 x MyTaq Red reaction buffer and a final primer concentration of 0.2 µM in a final reaction volume of 25 µL. Three sets of overlapping primers were used to amplify the entire VP2 region (1931 bp) as described previously, with minor modifications (Table 1) [12]. An initial denaturation step at 94 °C for 1 min, followed by 35–40 cycles of denaturation at 94 °C for 30 s, annealing at 50 or 55 °C for 30 s and extension at 72 °C for 30 s, ending with a final extension at 72 °C for 1 min, was used for DNA amplification. The PCR products were separated by electrophoresis on a 1% agarose gel (Bio-Rad Laboratories, Hercules, CA, USA) in 1× tris-acetate EDTA and visualized using SYBR Safe DNA (Invitrogen, Carlsbad, CA, USA). Sanger sequencing of positive PCR products was performed commercially (Macrogen, Seoul, Korea).

### 2.4. Sequence Analysis

VP2 sequences were edited, assembled and aligned using the ClustalW algorithm in Geneious Prime (Biomatters Ltd., Auckland, New Zealand) (although alignment was uncontroversial with no gaps needed). Sequences were translated in MegaX, and amino acid substitutions compared to the ICTV reference FPV sequence (FVP-3 Genbank accession no. EU659111) were identified. To determine the evolutionary history of the outbreak sequences from Australia, New Zealand and Dubai, we performed a phylogenetic analysis including these sequences and representative VP2 sequences of FPV and CPV taken from GenBank. In addition, we included an FPV VP2 sequence obtained from tissues of a healthy feral cat from Tasmania, Australia, sampled in 2010 (Genbank accession no. MN603976), for comparison. This resulted in a total data set of 205 sequences, 1774 nt in length. Phylogenetic analysis of these was performed using the PhyML program [13] and employing the GTR+I+Γ model of nucleotide substitution and a combination of Nearest-Neighbor Interchange (NNI) and Sub-tree Pruning & Re-grafting (SPR) branch swapping. Nodal support was assessed using SH-like branch support. Finally, the five CPV sequences included were used as an outgroup clade to root the tree.

### 2.5. FPV Viral Load Determination by qPCR and Multiplex PCR for Faecal Co-Pathogens

An FPV strain identical to that used in a commercially available MLV FPV vaccine was detected in faecal samples from New Zealand cases. Subsequently, qPCR was performed to determine FPV viral loads in DNA extracts of mesenteric lymph nodes and tissues from these cases using a TaqMan qPCR assay as described previously [14]. Faecal samples containing vaccine virus were also submitted to a commercial laboratory for batch testing on a multiplex qPCR panel to detect potential faecal co-pathogens including Feline coronavirus, *Tritrichomonas foetus*, *Clostridium perfringens*, *Giardia* spp, *Salmonella* spp, *Campylobacter jejuni*, *Campylobacter coli* and *Toxoplasma gondii* (Idexx Pty Ltd.).

### 2.6. Samples for Histological Examination

Tissues (duodenum, jejunum, colon, liver, spleen, kidney, mesenteric lymph node, heart, lung, pancreas, brain and/or bone marrow) from 11 representative Australian FPL cases that had died or been euthanised, ranging in age from 8 weeks to 12 months, including one from Melbourne in 2014 and 10 from Sydney in 2017 and 2018, were available for histological examination. Tissues (intestine, +/− mesenteric lymph node) from six suspected cases of FPL from New Zealand were also examined. All tissues were collected post-mortem and animals were not euthanised for the purposes of this study.

## 3. Results

### 3.1. Australian Cases

#### 3.1.1. Australian Cases—Outbreak Sites, Case Numbers and Distribution

**Figure 1:**
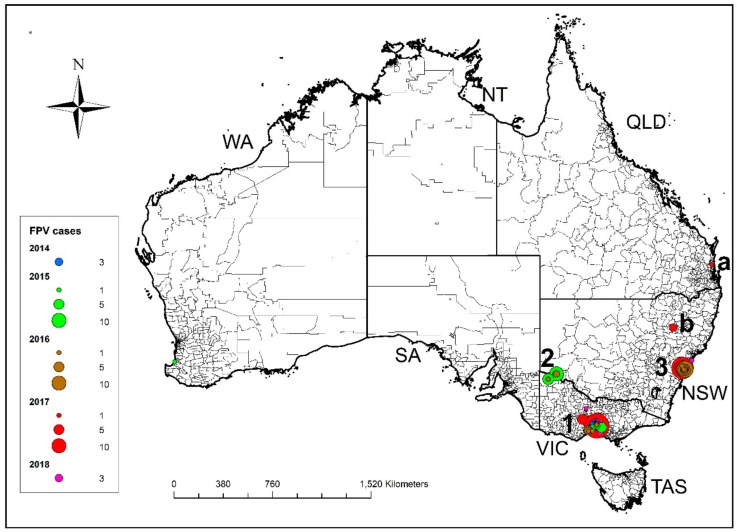
Distribution of cases of FPL reported in Australia between 1 January 2014 and 31 August 2018 by region and year of diagnosis. The first two outbreaks in Victoria occurred in 2014 in Melbourne (1) and in 2015 in Mildura (2). The third outbreak occurred in Sydney in December 2016 (3). Isolated cases were detected as far north as Nambour in Queensland (a) and in north western NSW in Gunnedah (b).

Data were received from 610 cases of FPL diagnosed in Australia between January 2014 and September 2018 from 11 animal shelters, 2 rescue societies and 11 veterinary hospitals. Of the 610 cases, 87% were shelter-housed, 12% were privately owned cats presenting to veterinary hospitals, and 1% were from foster carers working with rescue societies without premises. The distribution of Australian cases by geographic region and year is shown in Table 2 and Figure 1.

**Figure 2:**
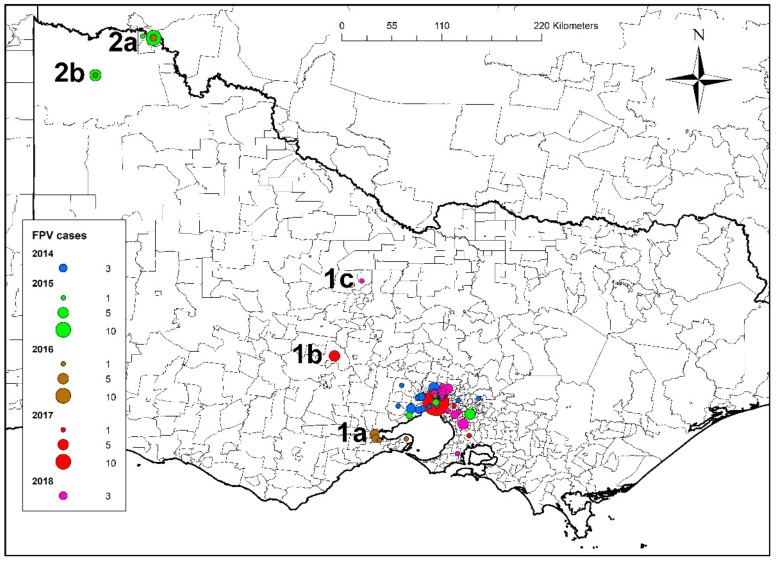
Distribution of cases in Outbreaks 1 and 2 from Victoria, Australia, showing expansion of the geographic area of FPL outbreaks originating in Melbourne from 2014 to 2018, including to Geelong in 2016 (1a), Bendigo in 2017 (1b) and Daylesford in 2018 (1c). Cases from the Mildura region outbreak were centred around the townships of Mildura (2a) and Benetook 2(b).

The first outbreak occurred in Melbourne, Victoria, between January and April 2014. A second outbreak occurred in Mildura, Victoria, some 560 km northwest of Melbourne, from February to April 2015, and a third outbreak occurred in Sydney, New South Wales (NSW), 1000 km northeast of Mildura and 900 km northeast of Melbourne from December 2016 to May 2017 (Figure 1). After the first recorded outbreak in each of these locations, between one and four recurrent seasonal outbreaks were documented, as well as isolated cases outside of the major outbreak regions, including in northwest NSW (Gunnedah) and southeast Queensland (Nambour) (Figure 1). The geographic area involved in recurrent annual seasonal outbreaks centred around Outbreak 1 from 2014 to 2018 expanded southwest in 2016, west in 2017 and northwest in 2018 (Figure 2). Similarly, there was expansion of the area involved in recurrent seasonal outbreaks centred around Sydney from 2016 to 2018, to involve the Central Coast region of NSW (Tuggerah and Wyong), 100 km north of Sydney (Supplementary Materials Figure S1).

**Figure 3:**
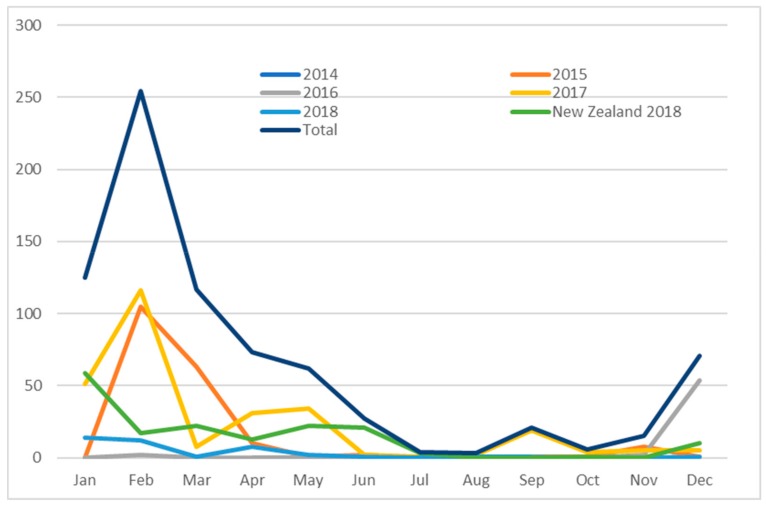
Seasonal distribution of Australian cases identified from 2014 to 2018 and of cases diagnosed in New Zealand in 2018.

The distribution of cases varied with month of presentation and is shown in Figure 3. Frequent transfer of cats in Victoria between shelters in Mildura (Outbreak 2 site) and Melbourne (Outbreak 1 site) suggested direct spread of the infection between these regions. Cats from two private shelters in Mildura were transported for rehoming to a shelter in Melbourne in 2014 and 2015.

#### 3.1.2. Australian Cases—Vaccination Protocols and Coverage

Two of five shelters in NSW did not vaccinate cats while under their care. The other three shelters vaccinated healthy cats on admission using an inactivated FPV vaccine, whereas sick cats with suspected viral respiratory tract infections were not vaccinated until their clinical signs resolved. None of the three shelters in Mildura vaccinated cats. Of the three shelters in Melbourne, one did not vaccinate cats, while two vaccinated cats on admission, one using an inactivated vaccine and the other an MLV vaccine. All shelters that used vaccinations gave a primary course to kittens starting at a minimum age of 6 weeks, then every 3 to 4 weeks, finishing at 12 to 14 weeks of age, while adult cats were given two vaccinations one month apart. Both rescue societies, one in NSW and one in Victoria, accepted unvaccinated cats and kittens for adoption from municipal pounds (shelters), that were fostered until adoption. For 177 shelter-housed cats, the median time from admission to diagnosis of FPL was 9 days (IQR 0–17; range 0–61 days).

Of the 610 cats diagnosed with FPL, 528 (87%) had never been vaccinated or had an unknown vaccination history. Sixty-five of the 82 (79%) cats that had received at least one vaccination were <16 weeks of age at the time of their last vaccination. For 17 cats ≥16 weeks of age at the time of last vaccination, the vaccination-to-disease interval was >10 days for 4/5 cats vaccinated with ≥2 inactivated vaccines and >7 days for 7/11 cats vaccinated with at least one MLV vaccine; vaccine type was unknown for one cat. For all vaccinated cats, the median time from last vaccination to diagnosis was 7.5 days (IQR 2–11; range 1–109 days).

Age, sex and breed data were available for 399 FPL cases. The median age at diagnosis was 10 weeks (IQR 7–20 weeks; range 3 weeks–96 months). The frequency distribution of age at diagnosis is shown in Table 3 Males (49%) and females (51%) were equally represented among FPL cases. Most cats (98%) were domestic crossbred (domestic shorthair or domestic longhair). Data on specific clinical signs were available for 153 cats. The most common clinical signs were diarrhoea (54%), lethargy (48%), vomiting (36%), anorexia (27%), weight loss or failure to gain weight (14%) and sudden death (10%). Diarrhoea was reported to be haemorrhagic in 13 of 83 cats (16%) with diarrhoea. Overall mortality for all 610 cats was 95%, noting that cases diagnosed in shelters were routinely euthanised at diagnosis to contain the infection.

### 3.2. Case data—New Zealand Cases

Two FPL outbreaks occurred in Wellington, New Zealand, in December 2016 and January 2018. The seasonal distribution of cases in the second outbreak is shown in Figure 3. In total, 365 cases of FPL were diagnosed, including 167 in the first outbreak and 198 in the second. During both outbreaks, 8% of feline admissions to the shelter were diagnosed with FPL. The median age at diagnosis was 9 weeks (IQR 6–14 weeks, range 2–105 weeks). At diagnosis of FPL, 32% of cats were unvaccinated, 58% had received one vaccine, 8% had been vaccinated twice, and 2% had been vaccinated three times. The median time since the last vaccination was 7 days (IQR 4–11.5 days, range 1–36 days). The median time from shelter admission to diagnosis was 7 days (IQR 0–14 days, range 0–28 days).

Of the nine cases for which clinical samples were available for PCR and sequencing, all cats had a positive faecal CPV antigen test result (FASTest Parvo Strip, Megacor Hoerbranz, Austria) and were euthanised at diagnosis. All cats had been vaccinated on admission using an MLV FPV vaccine (Felocell 3 or Felocell 4, Zoetis Pty. Ltd.). Clinical data are presented in Table 3.

### 3.3. Case Data—UAE Cases

Fourteen cases of FPL from Dubai, UAE, were diagnosed in domestic crossbreds with positive faecal CPV antigen test results. All cats were strays or strays that had been recently adopted, and all were unvaccinated, except an 18-month-old cat that had received a primary course of kitten vaccinations and a booster vaccination at 12 months. Clinical signs included vomiting, diarrhoea and lethargy, and the median age at diagnosis was 4 months (range 1–18 months). All cats were treated, and outcomes were known in 12 cases, of which 8 died or were euthanised, and 4 survived.

### 3.4. Histopathological Findings

#### 3.4.1. Histopathological Findings—Australian Cases

**Figure 4:**
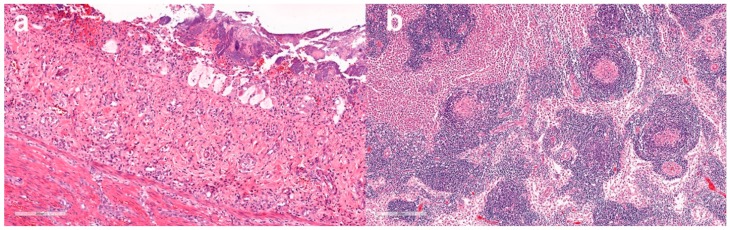
Case 111, 16-week old shelter-housed cat from Sydney, Australia, with diarrhoea, vomiting and fever, diagnosed with FPL. (**a**) Intestine—loss of villi and intestinal crypts of Lieberkuhn and collapse of proprial stroma with associated bacterial colonies on the luminal surface. Haematoxylin and eosin (HE) staining, scale bar 200 µm. (**b**) Mesenteric lymph node—lymphoid depletion is accompanied by accentuation of reticuloendothelial architecture with amorphous eosinophilic material within the centres of depleted follicles. HE. Scale bar 300 µm.

In sections of duodenum and jejunum from the 11 representative cases, there was a mild to severe necrotizing lymphoplasmacytic-to-suppurative enteritis, characterized by variable blunting, fusion and loss of villi, collapse of the lamina propria, loss of crypts and replacement by abundant coccobacilli and necrotic debris. The remaining crypts were often ectatic, with variable numbers of crypt abscesses (Figure 4). Evidence of multifocal crypt regeneration was also variably present. The submucosa and tunica muscularis were variably expanded by congestion, fibrin, oedema, haemorrhage, perivascular lymphocytes and plasma cells, macrophages exhibiting erythrophagocytosis, occasional neutrophils and rare eosinophils.

In sections of mesenteric lymph nodes, the most common finding was multifocal follicular and paracortical lymphoid depletion with accentuation of the reticuloendothelial architecture (Figure 4). Depleted lymphoid follicles were small, poorly populated and contained central accumulations of hyalinized eosinophilic material (follicular hyalinosis) with variable numbers of tangible body macrophages. The subcapsular and medullary sinuses were expanded by erythrocytes, oedema fluid and moderate to markedly increased numbers of macrophages, some exhibiting erythrophagocytosis.

Mild-to-moderate multifocal lymphoid depletion was also apparent in splenic sections. Extramedullary haematopoiesis characterized by the presence of myeloid and erythroid precursors was variably present within spleen and lymph nodes.

Sections of bone marrow examined were either hypocellular, characterised by increased numbers of, predominantly, adipocytes and extravasated erythrocytes interspersed with very low numbers of erythroid and myeloid precursors and megakaryocytes, or hypercellular, with increased numbers of myeloid and erythroid precursors. Some sections of the liver demonstrated hepatocellular atrophy and congestion, with minimal periportal mononuclear infiltrates. Among the sections of myocardium examined, there were no significant lesions.

#### 3.4.2. Histopathological findings—New Zealand cases

**Figure 5:**
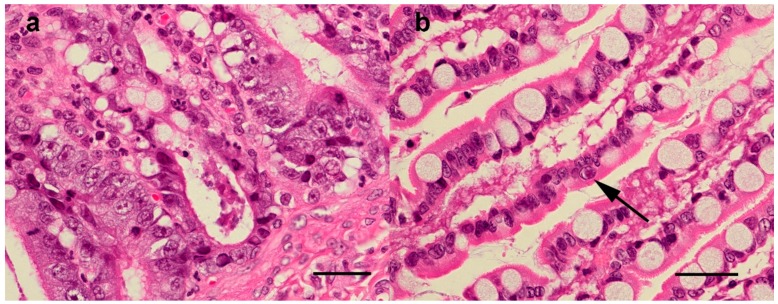
Small intestine, case 221, HE staining, scale bar 50 µm. (**a**) 600× Crypt epithelial cells are degenerating or necrotic and often sloughed into crypt lumens, (**b**) 1000× a villous epithelial cell contains an amphophilic intranuclear inclusion body which marginates the chromatin (arrow).

Histopathology of the small intestine was performed in six cases, including five in which samples were collected for PCR and sequencing (Table 4). In four of these, lesions of mild enteritis (*n* = 3) or fibrosis of the lamina propria (*n* = 1) were present. In one case (221, Table 3), histological lesions were typical of FPL (Figure 5), with multiple sections of jejunum and ileum showing mild multifocal loss and replacement of crypts by karyorrhectic and cellular debris (necrosis) and low-to-moderate numbers of neutrophils within the lamina propria. Low numbers of the remaining crypts were variably lined by degenerate or necrotic epithelium with intraluminal degenerate neutrophils, debris and sloughed necrotic epithelial cells (crypt abscesses). Neutrophils frequently invaded the remaining crypt epithelium. Rare epithelial cells contained 4–6 μm-diameter polygonal-to-ovoid, eosinophilic-to-amphophilic intranuclear inclusion bodies that occasionally marginated the chromatin. There was moderate multifocal crypt regeneration, and crypts were frequently lined by large epithelial cells with vesicular nuclei that were piled up or in mitosis. Intestinal lymphoid tissue appeared mildly depleted. The morphological diagnosis was moderate diffuse acute neutrophilic enteritis with crypt necrosis, epithelial regeneration and rare intranuclear viral inclusions. These findings were consistent with FPV/CPV infection. (Table 4). Of the cases where sections of mesenteric lymph nodes (*n* = 3) or thymus (*n* = 1) were available, lesions of mild follicular lymphoid depletion were present in one case, which was the same animal with small intestinal lesions consistent with FPV (case 221). Affected cortical follicles were small and poorly populated.

Histopathology was also performed on intestine and mesenteric lymph nodes from one unvaccinated cat with clinical signs of FPL, but for which samples were not collected for PCR and sequencing. Changes in the duodenum were similar to but more severe than those described above in case 221, including crypt necrosis and abscessation, neutrophilic infiltration of the lamina propria and the presence within epithelial cells of intranuclear inclusion bodies which variably marginated the chromatin. There was moderate lymphoid depletion in mesenteric lymph node tissue. These findings were also consistent with FPV/CPV infection.

### 3.5. VP2 Sequencing and Phylogenetic Analysis

**Figure 6:**
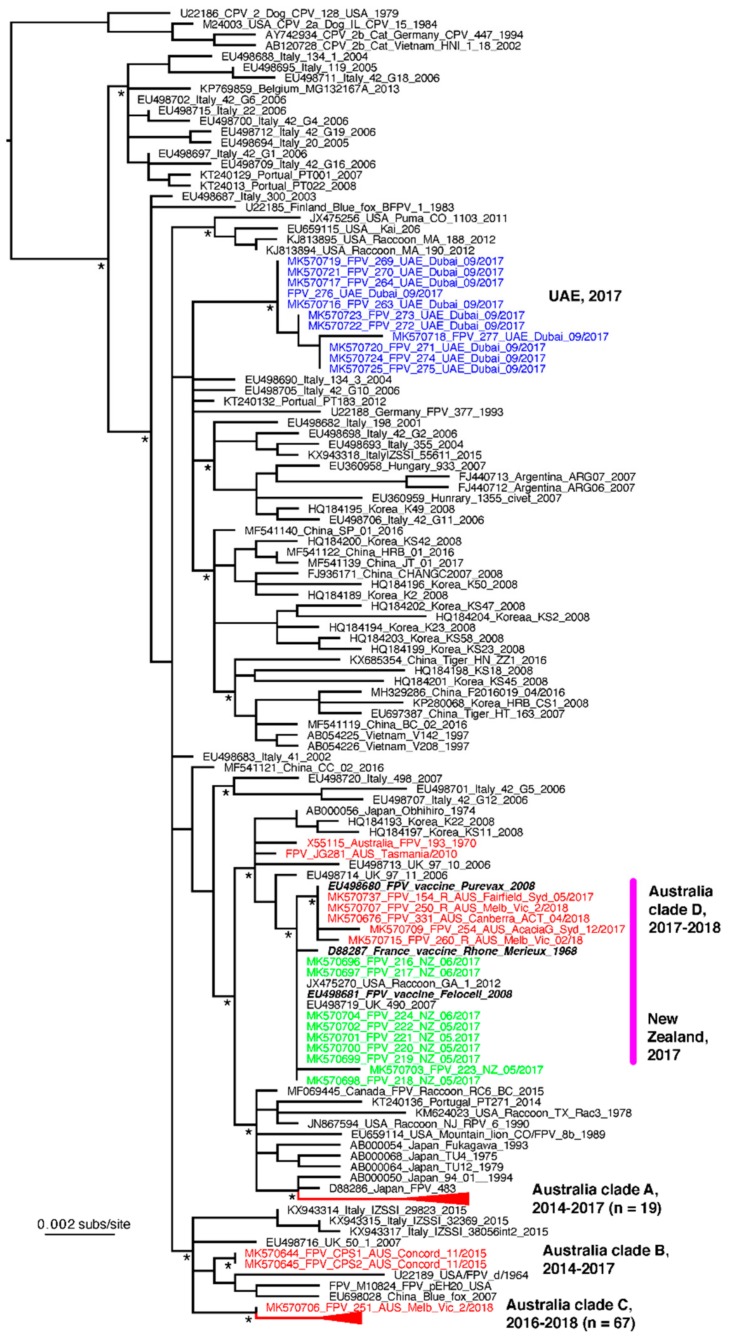
Phylogenetic relationships of FPV associated with outbreaks in Australia (red), New Zealand (green) and the UAE (blue). The virus sequences from the largest outbreak clades (A and C) have been collapsed for clarity, with the number of sequences in these clades shown in parentheses. FPV vaccine strains are shown in bold italic in the clades adjacent to the bold pink line. The tree is rooted on a small clade of canine parvovirus (CPV) sequences; * denotes SH-like branch support values of 1.0. Branch lengths are scaled to the number of nucleotide substitutions per site.

The entire VP2 region of *Carnivore protoparvovirus 1* was amplified from 113 suspected cases of FPL, comprising 93 from Australia, 11 from Dubai and 9 from New Zealand. Amino acid substitutions compared to the FPV reference strain are listed in Table 5. All VP2 sequences in this study were identified as FPV, with no cases attributed to CPV. Phylogenetic analysis of these VP2 sequences in comparison to a background global data set of FPV sequences revealed the presence of four distinct clades of Australian viruses (Figure 6). The first, denoted clade A, comprises 19 viruses (17 of which were identical) sampled from a wide geographic area (Melbourne, Geelong and Mildura) between 2014 and 2017. In contrast, Australia clade B comprised just two identical sequences sampled in NSW in 2015. These viruses were detected in the faeces collected from two cats in the same shelter in Sydney, NSW, in November 2015. Both cats had severe diarrhoea and tested positive for *Salmonella* spp. and *Carnivore protoparvovirus 1* on a multiplex faecal qPCR panel (Idexx Pty Ltd.). The largest Australian clade (C) comprised 66 viruses from NSW and one from Queensland, collected between December 2016 and February 2018. Viruses collected from cats in North Western NSW (Gunnedah) and South Eastern Queensland (Nambour) in 2017 were identical to those sampled from cats from four shelters and from privately owned cats in Sydney. Viruses from cats from the NSW Central Coast also fell into this clade. Finally, one sequence isolated from a cat from Melbourne in 2018 (FPV_251) was identical to viruses in clade C, with the exception of a mutation at position 694 (A694G) that resulted in an amino acid substitution I to V. This mutation was also present in viruses in Clade A.

### 3.6. FPV Viral Load and Concurrent Faecal Pathogens

A fourth (although not strictly monophyletic) clade (D) of viral sequences from Australian cats contained three VP2 sequences identical to that of an FPV vaccine strain (Purevax; Genbank accession no. EU498680) and two sequences that are very closely related to the vaccine strain. In one of these, from a 6-week-old kitten with acute vomiting and failure to gain weight, the vaccine strain was unlikely to be the cause of the cat’s clinical signs. FPL was suspected because a multiplex qPCR faecal panel was positive for *Giardia* spp. and *Canine protoparvovirus 1*. However, the cat, from Canberra in the Australian Capital Territory, had been vaccinated 16 days before with the same MLV vaccine strain. No littermates were affected, the cat recovered, and no other cases of suspected FPL were encountered in this region. Another sequence in this clade was from a 14-week-old stray kitten with vomiting, diarrhoea and failure to gain weight. The kitten, from a shelter in Melbourne, had been vaccinated 16 days before with the same MLV vaccine. It also tested positive on a multiplex qPCR faecal panel for *Giardia* spp. and *Canine protoparvovirus 1*. The third sequence was from an owned 8-week-old kitten in Sydney with acute onset vomiting and diarrhoea, vaccinated 3 days before (vaccine type unknown). Of the two closely related VP2 sequences, one (FPV_260) was from a 10-week-old kitten vaccinated 4 days before with the same vaccine strain, which presented for diarrhoea, vomiting and lethargy. It also tested positive for *Giardia* spp. on a faecal antigen test. The other (FPV_254) was from an unvaccinated stray 8-month-old cat that presented with vomiting, diarrhoea and anorexia and tested positive on a faecal CPV antigen test. Finally, two other Australian sequences—FPV 193/170 and Tasmania/2010—did not fall into a distinct clade but were relatively closely related to each other and to the vaccine cluster (clade D).

All 11 VP2 sequences from cats from Dubai fell into a single clade containing four unique sequence types and which was not clearly related to any other of the FPV sequences. In contrast, those viruses sampled from New Zealand in 2017 were either identical (eight of nine animals) or clearly very closely related to a vaccine strain (Felocell, Zoetis Pty Ltd.) and taken from cats with a history of recent vaccination. The single sequence that was not identical to that of the vaccine strain exhibited just two nucleotide substitutions, one of which was non-synonymous (E155K) (Table 4).

The results of the qPCR analyses to quantify FPV viral load in faeces of NZ cats and to detect faecal co-pathogens are presented in Table 4**.** Medical records were obtained from two unvaccinated kittens with FPL from Australia, where serial monitoring of FPV faecal shedding using qPCR was performed on pooled faecal samples after diagnosis. Testing was done at a single commercial laboratory, and results were reported as positive or negative (Table 6)**.**

## 4. Discussion

An understanding of the drivers of the re-emergence of FPL, the oldest known viral disease of cats, is essential to contain this fatal infection. We provide strong evidence of multiple outbreaks of FPL in three countries. There are no previous published reports of FPL outbreaks in any of these regions for comparison, although there is anecdotal first-hand experience of FPL among veterinarians practicing in Australia from the late 1960s to the mid-1970s, before commercial vaccines were used routinely [1]. Despite the widespread circulation of CPV in Australia, this virus was not detected in any samples tested in these FPL outbreaks. Canine parvovirus causes approximately 5% of FPL cases globally, but these have been confined to sporadic individual cases, and there are no reports of FPL outbreaks caused by CPV in multi-cat environments [8,15,16]. Whether this is due to viral or to host factors is currently unknown and warrants further investigation.

A single FPV lineage (clade A) was responsible for disease in both outbreak regions in Victoria, Australia, even though these are geographically distant (550 km) from each other. Phylogenetic analysis revealed the closest related virus was taken from a domestic cat in Japan in 1994 (GenBank accession no. AB000050), although it is likely that a closer ancestral virus exists but has not been sampled here. The 100% sequence identity of VP2 sequences from cases in Mildura and Melbourne is consistent with the epidemiological investigation, which found evidence supporting direct viral spread through transport of unvaccinated cats for adoption in 2014 and 2015. The nonenveloped virions of FPV, shed in high titres in all excretions of infected animals, are environmentally resilient and remain viable in infected premises such as shelters for over a year [17,18]. Indirect transmission by fomites is a major mechanism of parvovirus transmission among cats and dogs and was likely a crucial contributing factor to the canine global CPV pandemic in the late 1970s [19].

Unexpectedly, the FPL outbreak in Sydney, NSW, was not caused by spread of the Victorian strain, but rather by a separate clade (C) comprising a relatively distinct virus [20,21]. This finding, together with the detection of additional Australian FPVs (Clades B and D), is consistent with multiple independent virus entries into Australia, including the virus from an unvaccinated cat (clade D), which was seemingly derived from an FPV vaccine strain. Three recently vaccinated cats, in which the same vaccine virus was detected (clade D), likely did not have FPL, including two with *Giardia* co-infections initially diagnosed with FPL from a positive commercial faecal qPCR assay. MLV vaccine virus shed in faeces can be detected in qPCR assays and CPV faecal antigen tests [22,23]. Quantifying viral loads can assist in the discrimination of vaccine and field strains. However, quantification data were not provided by the commercial laboratory for these two cases. CPV vaccine viral loads in recently vaccinated dogs are 4- to 5-fold lower than those of dogs infected with field strains [24]. Similarly, a recent report found several recently vaccinated cats had low FPV viral loads (<1.38 × 10^2^ copies/mg of faeces) over a 28-day period of surveillance [23].

Before this investigation, only one FPV VP2 sequence from Australia had been deposited in the GenBank database (FPV 193/70, GenBank accession no. X55115). The evolutionary relationships with FPV 193/70 cannot be inferred reliably, since the isolate, obtained from a 3-month-old cat in Melbourne with peracute fatal FPL, had been serially passaged in vitro before sequencing in 1990. Interestingly, FPV 193/70 falls notably close to a clade of vaccine viruses in our analysis. In addition, we generated the VP2 sequence of an FPV strain detected in a healthy feral cat from Tasmania in 2010, which was genetically distinct from outbreak strains in this study and also fell into clade D, suggesting a vaccine-related origin. These findings, together with a report of FPV detection in the bone marrow of a healthy feral cat from Melbourne in 2012 [25], provide evidence that FPV has been circulating widely in Australia, at least since the 1970s, although the viruses are phylogenetically unrelated to the 2015–2018 outbreaks.

One possible explanation for the multiple independent FPV introductions described here is that FPV viruses circulating in the feral cat population may occasionally spill over into domestic cats. In comparison to the owned cat population, estimated at 3.8 million [26], some 2.1 to 6.3 million feral cats (free-roaming, unowned, unsocialised cats) inhabit Australia [27]. An FPV seroprevalence of 79% among feral cats in Victoria and NSW, documented in 1981, provides evidence of widespread FPV exposure [28]. Viral introductions could also have been anthropogenic, for example through fomite spread during international travel.

We were also able to analyse sequence data from a contemporaneous FPL outbreak in the UAE in 2018. This is the first study to characterise full-length VP2 sequences from the Middle East. Viruses most closely related to the UAE viruses, which formed a phylogenetically distinct lineage, were from a range of locations in Europe, Asia and the Americas, but the long lineage leading to the UAE viruses suggests that we have not yet sampled their ultimate ancestor.

The VP2 sequence data from cases analysed from a third country with contemporaneous FPL outbreaks showed that the NZ FPV strains segregated with FPV vaccine viruses. The virological findings from these cases yet again highlight the difficulty of diagnosing FPL in cats that have recently received MLV vaccines, as well as raising important questions about the potential capacity of reversion to virulence of MLV vaccines. The diagnosis of FPL in cases from NZ was initially based on consistent clinical signs and a positive faecal antigen test. Point-of-care immunochromatographic assays designed to detect CPV faecal antigen also detect FPV, so are used in practice for the diagnostic investigation of cats with clinical signs consistent with FPL [22]. However, faecal antigen tests detect both field and vaccine strains of FPV and CPV. In one study evaluating three commercial CPV faecal antigen kits, 20% of cats vaccinated against FPV in the preceding 14 days returned positive test results [29].

The sensitivity of faecal antigen tests for the detection of parvoviruses is influenced by viral load in faeces, as well as by the presence of gut antibodies that can sequestrate viral particles and prevent binding to the test antibody [30]. CPV vaccine viral loads in recently vaccinated dogs are usually below the limit of detection of antigen tests, ranging from 1.48 × 10^0^ to 2.5 × 10^4^ copies/mg of faeces in one study [24] and from ≈10^0^ to 7.50 × 10^5^ copies/mg of faeces in another [31]. The lower limit of detection of faecal antigen tests for CPV in dogs is 10^5^ to 10^6^ viral DNA copies per mg of faeces [32]. Low titres of gut FPV antibodies in the New Zealand cats could explain the positive faecal antigen test results in those cats with low faecal viral loads.

All but one of the VP2 sequences obtained from the New Zealand cases were identical to the FPV vaccine strain that the cats had been inoculated with. Co-infection with other pathogens including Feline coronavirus or *Giardia* spp. could account for the clinical signs in five of these cases, which, although consistent with FPL, were non-specific. However, in one cat, high loads of vaccine strain virus were detected concurrently in lymph node and faeces (FPV_221, Table 3), which is more consistent with loads attained by pathogenic virus strains than with those attained by vaccine virus strains. Further, unequivocal support of the diagnosis of FPL in this case was obtained from histological examination of the intestine, where lesions pathognomonic for FPL were identified (Figure 5). An FPV-vaccine strain was detected in this cat, and the sequencing chromatogram showed no evidence of a coinfecting FPV or CPV. However, deep sequencing of the sample with examination of the metatranscriptome or metagenome would be required to rule out co-infection with other pathogenic viruses not included in the qPCR test panel. An alternative explanation is that there was reversion to virulence of the vaccine strain, although this has not been described previously in cats.

Molecular analysis of faeces from dogs displaying signs of acute gastroenteritis shortly after CPV vaccination ruled out reversion to virulence of CPV MLV, since co-infection with field strains of CPV or other pathogens including Canine coronavirus, Canine distemper virus and *Isospora canis* was detected [33]. In three pups in which only the vaccine virus was detected, CPV vaccine loads were lower than those associated with enteritis from CPV field strains, ranging from 1.03 × 10^3^ to 1.78 × 10^5^ DNA copies/mg of faeces [33].

The re-emergence of FPL in two Australian states in relatively quick succession over a three-year period, caused by two distinct FPV lineages, raises the question as to why this disease is re-emerging now, after decades of apparent quiescence. Animal shelters are conducive environments for pathogen emergence or re-emergence because of a large number of susceptible hosts living in a confined area. This is exacerbated by factors including young age, immunological naivety or immunosuppression, close contact and co-morbidities such as heavy parasite burdens. Similarly, suboptimal biosecurity protocols favour pathogen persistence and fomite transmission [34].

Failure to vaccinate or inadequate or inappropriate vaccination in shelter-housed cats were major contributors to the re-emergence of FPL in Australia. Before these outbreaks, over half of the shelters from which cases were derived did not administer FPV vaccinations. In general, FPV vaccines are a highly effective tool in parvovirus control. For example, protective FPV antibody titres were measured in 95% of vaccinated cats in a field study of adult cats after MLV vaccination, of which 64% had pre-existing protective titres [35]. In addition, viral challenge studies of FPV-vaccinated cats usually show vaccine efficacy >90% [36]. Although state legislation mandates vaccination of owned cats in commercial boarding facilities in NSW and Victoria, vaccination is not enforced for cats admitted to private or municipal animal shelters, until they are sold for rehoming. Most shelters that did vaccinate, completed the primary course of vaccinations in kittens by 12 to 14 weeks of age and used inactivated vaccines, in contravention of the current guidelines. The World Small Animal Veterinary Association (WSAVA) Vaccination Guidelines recommend the use of MLV vaccines in shelter-housed cats for rapid induction of long-lasting humoral immunity [22,37]. A minimum age of 16 weeks for the final vaccination in the primary course is recommended, based on the failure of 45% of kittens to seroconvert after vaccinations at 8 and 12 weeks of age with the commercial FPV MLV vaccination [37,38]. This immunity gap relates to persistent maternally derived antibodies (MDA) in kittens, which decline below protective titres yet effectively neutralize the vaccine virus and thereby prevent seroconversion [38]. Persistent MDA would explain the presence of vaccination non-responders in our study that were <16 weeks of age at the time of diagnosis of FPL, highlighting that vaccination alone cannot prevent disease in environments where susceptible kittens are exposed to FPV.

Among the 21% of vaccination non-responders that were older than 16 weeks, some were likely exposed to FPV prior to seroconversion, either before or after admission to the shelter, as indicated by the median vaccination-to-diagnosis interval of 7.5 days. The incubation period for FPL is between 2 and 10 days [17]. Other reasons for apparent vaccine failure include persistence of MDA for >16 weeks in kittens born to queens with high anti-FPV antibody titres or failure to mount an immune response to vaccination because of immunodeficiency (acquired or genetic), incorrect vaccine administration or storage errors [35,38].

The median time from shelter admission to diagnosis of FPL was 9 days. The minimum holding time for unidentified cats in municipal shelters in the states of Victoria and NSW is 7 days, after which cats can be euthanised or rehomed (Domestic Animals Act 1994, Companion Animals Act 1998). Changes in euthanasia practices resulting in longer length of stay (LOS) may have contributed to a cumulative increase in selection pressure for FPL re-emergence, especially in shelters where vaccination was not practiced. The likelihood of exposure to infectious diseases increases within a shelter environment with increasing LOS [39]. National data from Royal Society of Prevention to Cruelty (RSPCA) shelters show a progressive decline in euthanasia rates from 74% in 1995 to 27% in 2018 [40,41]. Similar declines in euthanasia have occurred in shelters in other countries such as Canada [42].

A seasonal pattern of FPL was identified in Australia and New Zealand, with an annual peak in case numbers from December to May. This pattern may reflect waning maternal immunity lagging two to three months behind the peak kitten births. Similar seasonality of FPL outbreaks was reported in the Northern Hemisphere, where seasons are inverted; case numbers peaked from July to December and were correlated with an influx of kittens born annually in spring and waning of maternal immunity over the subsequent two to three months [43].

The duration of faecal shedding of FPV, which is relevant to infection control, has been inadequately studied. This study demonstrates that virus can be shed in faeces for an extended period of up to 14 weeks from diagnosis. Faecal shedding of vaccine virus was detected by qPCR post vaccination for the duration of one four-week study [23]. Shedding of field CPV strains in dogs has been detected by qPCR for 45–50 days [44,45], while CPV vaccine virus was shed for 19–22 days on average [31,46]. Further studies are warranted in cats to further define the duration of faecal shedding of both field and vaccine strains of FPV detectable by qPCR.

## 5. Conclusions

Inadequate vaccination coverage was identified in the FPL outbreaks described here and was a major contributor to the re-emergence of this disease. These outbreaks highlight the importance of adherence to vaccination guidelines for shelters, recommended by professional bodies such as the WSAVA. Further research is needed to identify the duration of FPV shedding in naturally infected cats, as well as the potential for reversion to virulence of MLV vaccines.

## Figures and Tables

**Table 1:** Primer sequence and amplicon length for amplification of the VP2 region [12].

Primer	Sequence 5′–3′	Fragment Size (bp)
CPV2679-F	CCAGATCATCCATCAACATCA	853
CPV3511-R	TGAACATCATCTGGATCTGTACC	
CPV3381-F	CCATGGAAACCAACCATACC	736
CPV4116-R	AGTTAATTCCTGTTTTACCTCCAA	
555-F	CAGGAAGATATCCAGAAGGA	583
555-R	GGTGCTAGTTGATATGTAATAAACA	

**Table 2:** Number of Australian cases of feline panleukopenia (FPL) by year and region.

Year	NSW ^1^	QLD ^2^	VIC ^3^	WA ^4^	Total
2014	0	0	40	0	40
2015	3	0	188	1	192
2016	38	0	21	0	59
2017	211	1	66	0	278
2018	3	0	38	0	41
Total	255	1	353	1	610

^1^ NSW: New South Wales; ^2^ VIC: Victoria; ^3^ QLD: Queensland; ^4^ WA: Western Australia

**Table 3:** Frequency distribution of age at diagnosis for 399 cases of FPL.

			Cumulative	
Age	Frequency	Percent	Frequency	Percent
0–5 weeks	51	13	51	13
6–10 weeks	155	39	206	52
11–14 weeks	39	10	245	61
15–18 weeks	47	12	292	73
19–24 weeks	22	6	314	79
6–12 months	22	6	336	84
1–2 years	46	12	382	96
>2 years	17	4	399	100

**Table 4:** Summary of data obtained from New Zealand cases that had positive CPV faecal antigen test results and FPV VP2 sequence amplification using conventional PCR and/or histological findings consistent with FPL.

Case	Age (Weeks)	N ^1^	D ^2^	Clinical Signs	FPV Viral Copies/mg of Lymph Node	FPV Viral Copies/mg of Faeces	Faecal Co-Pathogens Detected on Multiplex PCR/Giardia Faecal Antigen Tests	Histological Findings—Small Intestine
216	10.5	2	15	Weight loss, diarrhoea	2.48 × 10^8^	4.12 × 10^3^	Feline coronavirus	NT
217	11	2	15	Diarrhoea	9.63 × 10^6^	6.79 × 10^3^	NT	NT
218	10.5	1	14	Dehydration, diarrhoea	2.87 × 10^7^	4.12 × 10^3^	*Giardia* spp.	Enteritis, acute, neutrophlic, mild
219	9.5	1	11	Diarrhoea	1.77 × 10^3^	3.4 × 10^3^	Feline coronavirus	NT, CBC WNL
220	13	1	7	Diarrhoea	8.22 × 10^9^	6.48 × 10^4^	Feline coronavirus	Mild diffuse fibrosis of lamina propria, CBC: mild lymphopenia
221	7	1	7	Conjunctivitis, weight loss	1.08 × 10^10^	1.27 × 10^8^	*Clostridium perfringens*	Multifocal crypt necrosis, crypt abscesses, lymphoid depletion, viral inclusion bodies
222	10	1	6	Diarrhoea	1.17 × 10^10^	1.32 × 10^6^	None	Enteritis, neutrophilic and eosinophilic, mild, acute
223	10	1	6	Diarrhoea	1.32 × 10^10^	NT	NT	Enteritis, plasmacytic, mild
224	18	2	8	Diarrhoea, sneezing	1.79 × 10^8^	8.02	Feline coronavirus	NT
88153	6	U	-	Vomiting, dehydration	NT	NT	*Giardia* spp.	Similar but more severe lesions, to case 221

^1^ N: Number of vaccinations received; ^2^ D: Days since last vaccination; CBC: complete blood count; NT: not tested; U: unvaccinated; WNL: within normal limits

**Table 5:** Amino acid strain substitutions in the VP2 region of strains of feline parvovirus (FPV) isolated from cats in Australia, New Zealand and the United Arab Emirates compared to the reference FPV strain nominated by the International Committee on Taxonomy of Viruses (ICTV) (FVP-3, EU659111).

Accession No.	Variant	Year	Amino Acid VP2 Location																					
			5	80	87	93	101	103	145	204	232	297	300	305	323	373	375	426	445	462	555	562	564	568
EU659111 ^1^	FPV ref	1967	A	K	M	K	T	V	I	I	V	S	A	D	D	D	D	N	T	P	V	V	N	A
MK570663	This study	2016–2018	•	•	•	•	•	•	•	•	•	•	•	•	•	•	•	•	•	•	•	•	•	•
EU498681 ^2^	Felocell *	2008	•	•	•	•	•	•	•	•	I	•	•	•	•	•	•	•	•	•	•	L	•	•
EU498680 ^3^	Purevax *	2008	•	•	•	•	•	•	•	•	I	•	•	•	•	•	•	•	•	•	•	L	•	•
MK570709	This study *	2017	•	•	•	•	M	•	L	•	I	•	•	•	•	•	•	•	•	•	•	L	•	•
MK570715	This study *	2018	•	•	•	•	V	•	•	•	I	•	•	•	•	•	•	•	•	•	•	L	•	•
MK570637 ^4^	This study	2015–2016	T	•	•	•	•	•	•	•	•	•	•	•	•	•	•	•	•	•	•	•	•	•
MK570646	This study	2015	T	•	•	•	•	•	•	•	•	•	•	•	•	•	•	•	I	•	•	•	•	•
MK570644 ^5^	This study	2015	•	•	•	•	I	•	•	•	I	•	•	•	•	•	•	•	•	L	•	•	•	•
MK570720 ^6^	This study	2017	•	•	•	•	•	•	•	V	•	•	•	•	•	•	•	•	•	•	•	•	•	•
MK570654 ^7^	This study	2017–2018	•	•	•	•	•	•	•	•	I	•	•	•	•	•	•	•	•	•	•	•	•	•
MK570710	This study	2017	•	•	•	•	•	•	•	•	I	•	•	•	•	N	•	•	•	•	•	•	•	•

* vaccine strain; • Amino acid identical to FPV reference EU659111; ^1^ MK570664, MK570667–MK570669, MK570677–MK570679, MK570684–MK570691, MK570693–MK570695, MK570707, MK570711–MK570713, MK570716, MK570717, MK570719, MK570721–MK570723, MK570726, MK570731, MK570732, MK570734–MK570736, MK570741–MK570744 and MK570746–MK570748 have identical amino acid sequences; 2 MK570696–MK570704 have identical amino acid sequences; 3 MK570707, MK570714, MK570737 and MK570676 have identical amino acid sequence; 4 MK570638–MK570643, MK570647–MK570653 and MK570749 have identical amino acid sequences; 5 MK570645 has an identical amino acid sequence; 6 MK570724, MK570725, MK570718 and MK570675 have identical amino acid sequences; 7 MK570655–MK570662, MK570665 and MK570666, MK570670–MK570674, MK570680–MK570683, MK570692, MK570705 and MK570706, MK570708, MK570727–MK570730, MK570733, MK570738–MK570740 and MK570745 have identical amino acid sequences

**Table 6:** Results of serial monitoring of faecal shedding of FPV in faecal samples from two cats diagnosed with FPL.

	CPV Faecal Antigen Test ^1^	Parvovirus Detection by Faecal qPCR ^2^			
	21.11.2016	07.01.2017	04.02.2017	07.03.2017	11.04.2017
**Result**	positive	positive	positive	positive	negative

^1^ Individual samples from both cats tested positive; ^2^ Faecal samples from both cats were pooled for testing at all time points.

## Supplementary Materials

The following are available online at https://www.mdpi.com/1999-4915/11/12/1155/s1, Figure S1: Geographic and temporal distribution of cases in Feline panleukopenia outbreaks in New South Wales Australia.
