# Supplementary figures and images for: Distinct Lineages of Feline Parvovirus Associated with Epizootic Outbreaks in Australia, New Zealand and the United Arab Emirates
Source: Viruses. 2019 Dec 13;11(12):1155. doi: 10.3390/v11121155 (PMC6950618; doi:10.3390/v11121155)

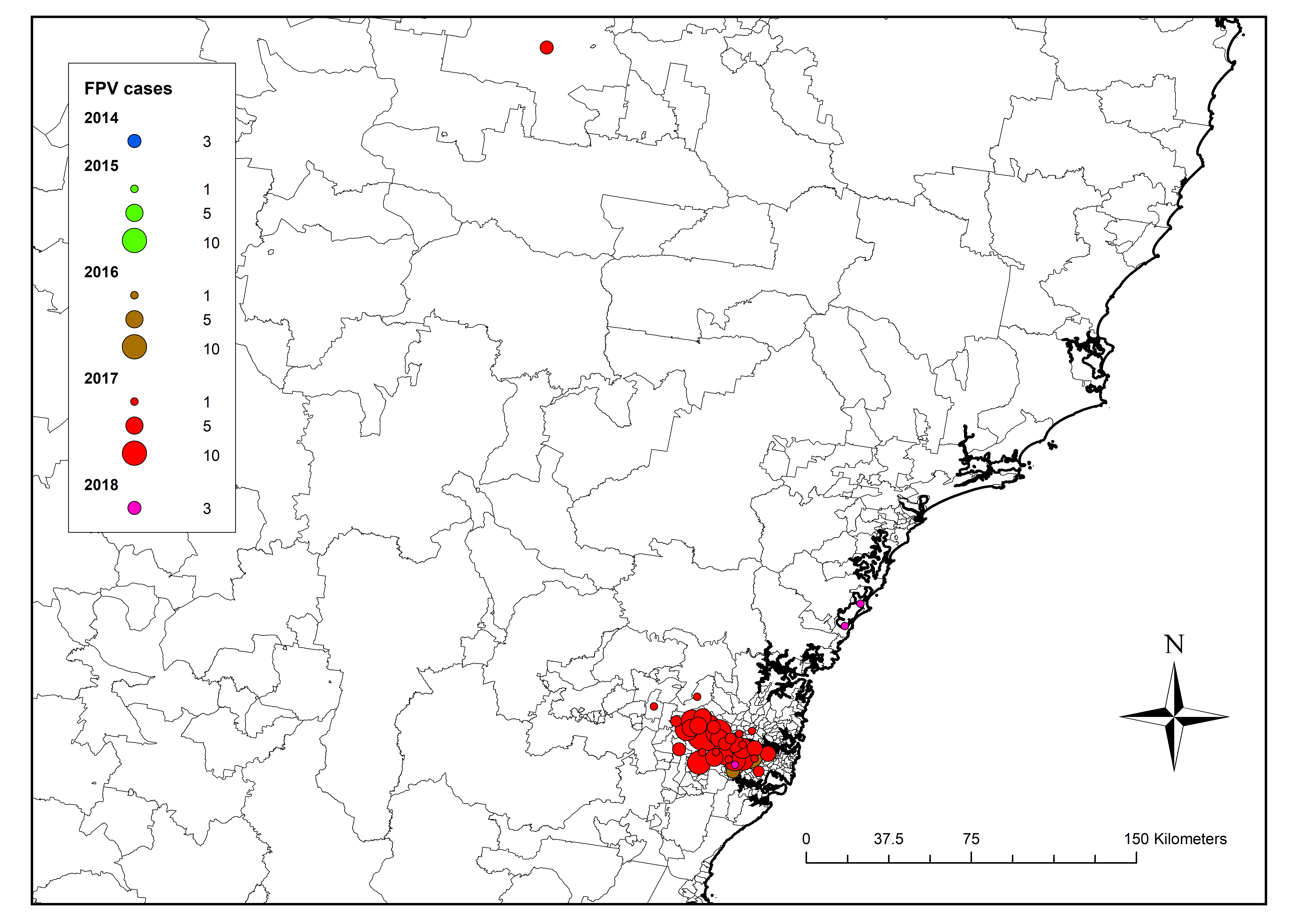

Supplement: Supplementary file 1 [file viruses-11-01155-s001.jpg]
